# *Tanacetum parthenium* enhances pentobarbital-induced sleeping behaviors

**Published:** 2020

**Authors:** Fatemeh Forouzanfar, Hamed Ghazavi, Mohammad Mahdi Vahedi, Kimia Tarrah, Zahra Yavari, Azar Hosseini, Azita Aghaee, Hassan Rakhshandeh

**Affiliations:** 1 *Neuroscience Research Center, Mashhad University of Medical Sciences, Mashhad, Iran*; 2 *Department of Neuroscience, Faculty of Medicine, Mashhad University of Medical Sciences, Mashhad, Iran *; 3 *Department for Population and Family Health, Vice Chancellery for Health, Mashhad University of Medical Sciences, Mashhad, Iran*; 4 *Health Promotion Research Center, Zahedan University of Medical Science, Zahedan, Iran*; 5 *Department of Pharmacology, School of Medicine, Zahedan University of Medical Science, Zahedan, Iran*; 6 *Pharmacological Research Center of Medicinal Plants, Mashhad University of Medical Sciences, Mashhad, Iran*; 7 *Medical Toxicology Research Center, School of Medicine, Mashhad University of Medical Sciences, Mashhad, Iran*

**Keywords:** Pentobarbital, PC12, Sleep, Tanacetum parthenium, Herbal medicine

## Abstract

**Objective:**

Sleep disorders are among the most common psychiatric and medical conditions. In the present study, the hypnotic effect of *Tanacetum parthenium *was studied in mice**. **

**Materials and Methods:**

The hydro-alcoholic extract (HAE) of *T. parthenium* and three fractions of it, namely water fraction (WF), ethyl acetate fraction (EAF), and *n*-hexane fraction (NHF), were intraperitoneally (ip) administrated to mice 30 min before injection of sodium pentobarbital (30 mg/kg, ip). Then, 30 min after administration of HAE, motor coordination (rota-rod test) was evaluated. Besides, LD_50_ of HAE was determined and the cytotoxicity of HAE was evaluated in PC12 cells using the MTT assay.

**Results:**

HAE 50-200 mg/kg increased the sleeping time. EAF was the only fraction which could prolong the sleep duration and decrease sleep latency. The LD50 value was 4.8 g/kg. The extract induced no cytotoxic effects in PC12 cell line.

**Conclusion:**

The results suggested that *T. parthenium* potentiates pentobarbital hypnosis without causing toxic effects. Probably, its effects are mediated by the components present in EAF of this plant.

## Introduction

Sleep disorders, such as insomnia, are a growing mental health problem, that significantly impact the quality of life and frequently cause poor memory, slower reactions, and emotional disturbances (Auld et al., 2017 ▶). Benzodiazepines as the most widely used therapeutics administered to treat sleep problems, were introduced into clinical practice in the 1960s (Ferreri et al., 2015 ▶; Riemann et al., 2015 ▶). 

Benzodiazepines increase the effect of the neurotransmitter gamma-aminobutyric acid (GABA) at the GABAA receptor, increasing inhibitor outputs to all major cell groups in the brainstem and the hypothalamus that promotes arousal. Various adverse-effects, such as dependence and tolerance have been associated with long-term use of benzodiazepines (Vinkers and Olivier, 2012 ▶; Ferreri et al., 2015 ▶). Consequently, there has been a growing demand for substances that could contribute to inducing sleep and improving its quality with less adverse-effects. *Tanacetum parthenium*, known as feverfew, is a daisy-like perennial plant from the family Asteraceae which is commonly found in gardens and along roadsides (Pareek et al., 2011 ▶). This herb had been prescribed by the Greek physician Dioscorides for “all hot inflammations”. Also, the plant is known as “featherfew” because of its feathery leaves (Pareek et al., 2011 ▶).

The plant has been used for treating various diseases including arthritis, earache, asthma, constipation, dermatitis, stomach ache, fever, headache, inflammatory conditions, spasms, menstrual disorders, swelling, and toothache (Jain and Kulkarni, 1999 ▶; Pareek et al., 2011 ▶). In traditional medicine, this plant has been used as an antipyretic, from which its common name is derived (Jain and Kulkarni, 1999 ▶; Pareek et al., 2011 ▶). In some traditional medicinal books, it is described that *T. parthenium *has sedative–hypnotic effects (khorasani, 1371 ▶). Previous studies showed that some components of this plant for example alpha-pinene derivatives may have sedative and mild tranquilizing properties (Pareek et al., 2011 ▶). The aim of this study was therefore to examine the sleep-prolonging action of *T. parthenium* hydro-alcoholic extract (HAE) and its fractions. Also, the safety of this plant was examined in neuronal cells by determination of LD_50_.  

## Materials and Methods

**Drugs and chemicals**

Flumazenil, sodium pentobarbital, penicillin-streptomycin, and 3-(4, 5-dimethyl thiazole-2yl)-2, 5-diphenyl tetrazolium bromide (MTT) were obtained from Sigma (USA). Tween 80 was obtained from Merck (Germany). Dulbecco’s Modified Eagle’s Medium (DMEM) and fetal bovine serum (FBS) were purchased from GIBCO (USA). Diazepam was bought from Chemidarou Company (Iran).

**Preparation of** ***T. parthenium*** **extract**

*T. parthenium* was collected from Mashhad (Khorasan province, Iran). The voucher specimen was prepared and deposited (No. 36396) in the School of Agriculture, Ferdowsi University of Mashhad, Mashhad, Iran and dried in a dark place at room temperature. 

The plant powder was subjected to extraction in a Soxhlet apparatus using 70% ethanol for 48 hr (Hosseini et al., 2018). Then, HAE was filtered and dried on a water bath. The dried remaining (30% w/w) was dissolved in saline that contained 1% (v/v) Tween 80. The HAE was stored at 4°C for several days until used. 

Control group received 1% Tween-80 in saline. For preparation of the fractions of HAE, a part of dried HAE was suspended in distilled water and transferred to a separator funnel. Using solvent-solvent extraction, it was fractionated by *n*-hexane or ethyl acetate to obtain ethyl acetate fraction (EAF) and *n*-hexane fraction (NHF), respectively. Then, The EAF and NHF were separated to obtain water fraction (WF). The resulting fractions were placed in a water bath to dry and working solutions were made up in saline and saline containing 1% Tween 80 for WF and EAF or NHF, respectively. The yield of WF, NHF and EAF were 70, 16 and 14%, respectively. 

**Animals**

Male albino mice weighting 20-30 g were maintained in a temperature-regulated environment (22±1°C) with 12 hr of light and 12 hr of dark and had free access to water and food. The research was conducted in accordance with the ethical guidelines of Mashhad University of Medical Sciences. Mice were randomly divided into 8 groups (n=8 per group). In the first experiment, to determine if HAE has a sleep-prolonging effect, the animals received vehicle (control group) and diazepam (3 mg/kg) as positive control or different doses of HAE. In the second experiment, to evaluate the most effective fraction of HAE, mice were treated with WF, EAF, NHF, or 1% Tween 80 (as the vehicle for EAF and NHF).

**Evaluation of pentobarbital-induced sleep**

Sleep time was evaluated in pentobarbital-induced mouse sleep model. A single dose of HAE 25, 50, 100 and 200 mg/kg, fractions of HAE 25 and 50 mg/kg, diazepam (3 mg/kg), or other vehicles was injected intraperitoneally (ip) into the mice. After 30 min, they received pentobarbital (30 mg/kg, ip) to induce sleep. Flumazenil (1 mg/kg) was given 30 min before diazepam or HAE. The onset of sleep is the time that animals stayed immobile and lost their righting reflex. The sleep latency was recorded as the time between administration of pentobarbital and onset of sleep (Hosseini et al., 2018 ▶).

**Rota-rod test**

The rota-rod test is a basic assessment tool for assessing motor coordination. The experimental procedure for learning and adaptation was performed during 3 consecutive days. On day 4, rats were placed on a rotating rod that accelerated smoothly from 4 to 40 rpm over a period of 5 min. The length of time they could maintain their balance on the turntable against the movement’s strength, was recorded. Then, the extract or vehicle was injected and after 30 min, they were placed again on the rota-rod (Hosseini et al., 2018 ▶).

**LD**_50_ **Determination**

Groups of 2 mice (eight groups) were used for determination of HAE LD_50_. Different doses (50-3200 mg/kg) of HAE extract were injected intraperitoneally into mice. Mortality rate was observed and recorded within a 24-hr period. The highest dose which did not kill any animal and the lowest dose which led to death of one mice per group were recorded. The average of these two doses was considered the median lethal dose (Akhila et al., 2007 ▶). 

**Neurotoxicity assessment**

The rat pheochromocytoma-derived (PC12) cells were seeded in 96-well plates and cultured for 24 hr in DMEM supplemented with 10% FBS, penicillin (100 units/ml) and streptomycin (100 µg/ml) at 37°C with 5% CO_2_. Then, the medium was replaced by a fresh one containing saline or HAE (50, 100, 200, 400 and 800 µg/ml). The cells were incubated for 24 hr under 5% CO_2_ atmosphere. Then, cell proliferation was determined using MTT assay as previously described (Hosseini, Sobhanifar et al. 2018 ▶). Briefly, the MTT (0.5 mg/ml) was added to culture medium and incubated for 2 hr. Then, the medium was discarded and the resulting formazan dye was dissolved using DMSO and the optical density of dye was measured at 545 nm. 

**Statistical analyses**

All data are expressed as mean±SEM. Statistical analysis was performed using one-way analysis of variance (ANOVA) followed by Tamhane’s T2 *post-hoc* test. 

Differences were considered significant at p<0.05.

## Results

**Effect of** ***T. parthenium*** **on sleep**

Sleeping time period in the animals received normal saline before pentobarbital was 23.5±1.7 min. As expected, the reference drug diazepam significantly increased the duration of sleep (42.8±1.2 min, p<0.001 vs. control). 

Duration of sleep in animals receiving HAE was increased to 37±2 min (p<0.05), 38±1.8 min (p<0.01), and 44.17±5.9 min (p<0.001), at doses of 50, 100, and 200 mg/kg, respectively.

**Figure 1:**
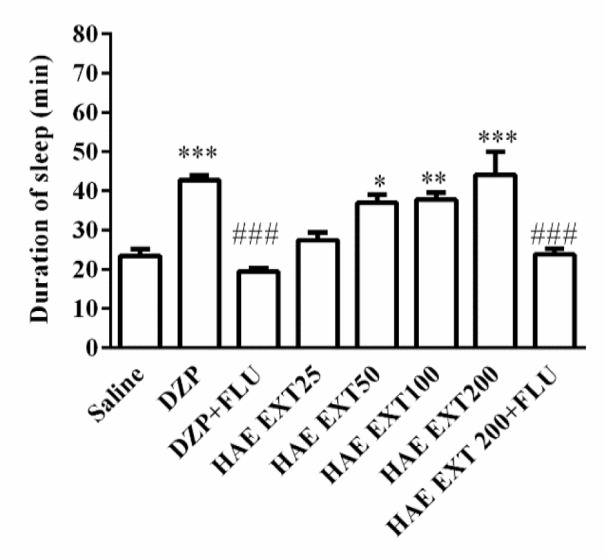
Effects of hydroalcoholic extract of *Tanacetum parthenium* on the duration of sleep. Different doses (50, 100, 150 and 200 mg/kg) of the extract or diazepam (DZP, 3 mg/kg) were administrated (ip) to mice 30 min before injection of pentobarbital (30 mg/kg, ip). Data are mean±SEM of 7 animals in each group. *p<0.05, **p<0.01, and ***p<0.001 vs. saline; ###p<0.001 vs. the same group without flumazenil (FLU, 2 mg/kg). DZP, diazepam; HAE EXT, hydro-alcoholic extract; FLU, flumazenil

 Flumazenil significantly reversed the sleep-prolonging effect of diazepam (Diazepam 42.83±1.2 and Diazepam+Flumazenil 19.50±.8 min, respectively) (p<0.001). Similarly, the effect of HAE on sleep duration was significantly inhibited by flumazenil (HAE 44.17±5.9 and HAE+Flumazenil 23.8±1.5 min, respectively) (p<0.001) (Figure 1).

**Figure 2:**
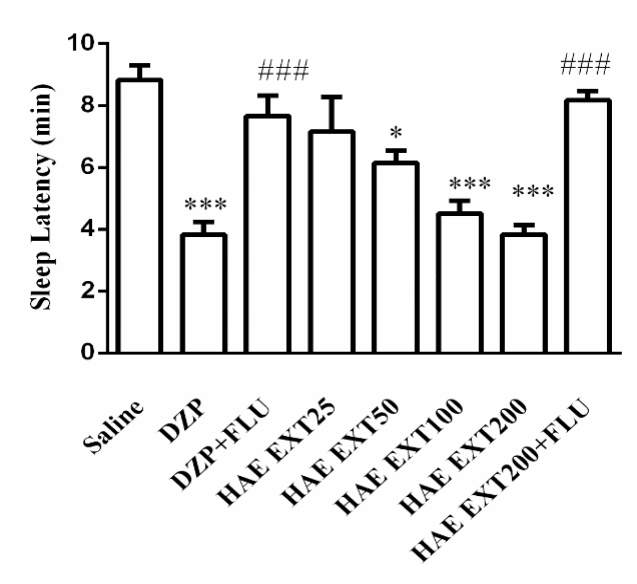
Effects of hydroalcoholic extract of *Tanacetum parthenium* on sleep latency. Different doses (50, 100, 150 and 200 mg/kg) of the extract or diazepam (DZP, 3 mg/kg) were administrated (ip) to mice 30 min before injection of pentobarbital (30 mg/kg, ip). Data are mean±SEM of 7 animals in each group. *p<0.05, and ***p<0.001 vs. saline; ###p<0.001 vs. the same group without flumazenil (FLU, 2 mg/Kg). DZP, diazepam; HAE EXT, hydro-alcoholic extract; FLU, flumazenil

 Diazepam (3.8±0.4 min, p<0.001) and HAE at doses of 50 (6.1±0.4, p<0.05) and 100 (4.5±0.4, p<0.001) and 200 mg/kg (3.8±0.3, p<0.001) significantly decreased the sleep latency as compared to the vehicle group (8.8±0.4 min). As it can be observed in Figure 2, pretreatment of mice with flumazenil reversed the effects of diazepam (7.6±0.63 min, vs. diazepam p<0.001) and HAE 200 mg/kg (8.1±0.3 min, vs. HAE p<0.001).

**Effect of** ***T. parthenium*** **extracts on sleep**

**Figure 3:**
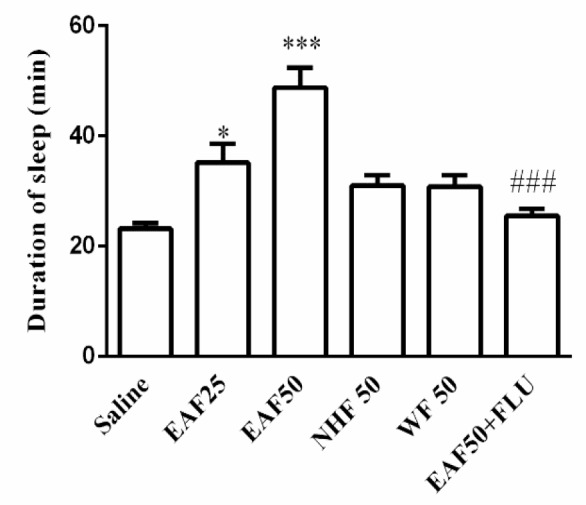
Effects of different fractions of *Tanacetum parthenium* extract on the duration of sleep. Saline containing 1% Tween, water fraction (WF), ethyl acetate fraction (EAF), or n-hexane fraction (NHF), was administrated (ip) to mice 30 min before injection of pentobarbital (30 mg/kg, ip). Data are mean±SEM of 7 animals in each group. *p<0.05, and ***p<0.001 vs. saline; ###p<0.001 vs. the same group without flumazenil (FLU, 2 mg/kg)

Among the fractions, EAF at the doses of 25 (35.1±3 min, p<0.05) and 50 mg/kg (48.83±3.6 min, p<0.001) was able to significantly increase the sleep time as compared to saline group. However, other fractions did not lead to statistically significant effects. Also, flumazenil reversed the prolonged hypnotic effects of EAF (EAF 25.5±1.3 vs EAF+Flumazenil 48.8±3.361 min) (p<0.001) (Figure 3).

**Figure 4:**
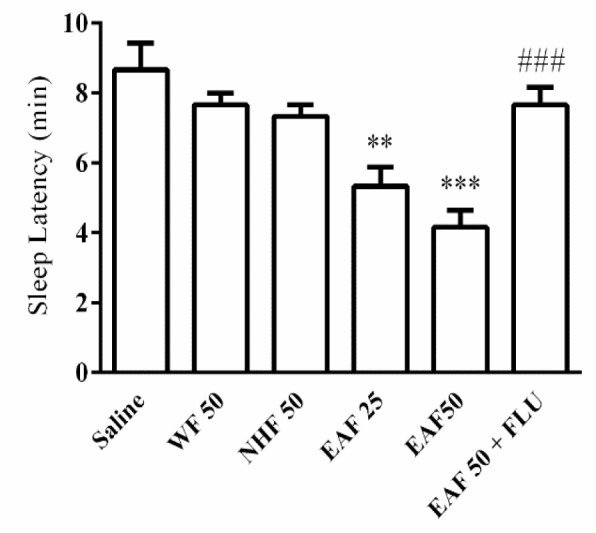
Effects of different fractions of *Tanacetum parthenium* extract on sleep latency. Saline containing 1% Tween (vehicle), water fraction (WF), ethyl acetate fraction (EAF), or n-hexane fraction (NHF), was administrated (ip) to mice 30 min before injection of pentobarbital (30 mg/kg, ip). Data are mean±SEM of 7 animals in each group. **p<0.01, and ***p<0. 001 vs saline; ###p<0.001 vs the same group without flumazenil (FLU, 2 mg/kg)

The sleep latency in vehicle group was 8.6±0.7 min. EAF at the doses of 25 (5.3±0.5 min, p<0.01) and 50 mg/kg (4.1±0.5 min, p<0.001) significantly decreased sleep latency. In flumazenil-treated mice, the effect of EAF on the sleep latency was significantly reversed (7.6±0.5 min vs 4.1±0.5 min, p<0.001) (Figure 4).

**Toxicity assessments**

**Figure 5:**
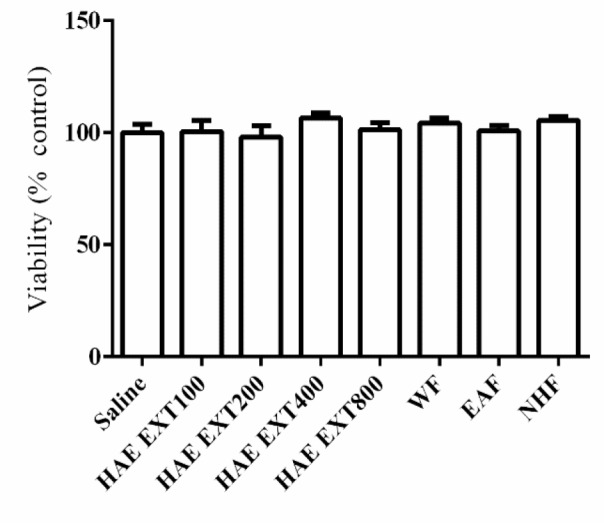
Effects of hydroalcoholic extract of *Tanacetum parthenium* on viability of neuronal PC12 cell. The cells were cultured for 24 hr in the medium containing saline, extracts (100-800 µg/ml), 800 µg/ml water fraction (WF), 800 µg/ml ethyl acetate fraction (EAF) and 800 µg/ml n-hexane fraction (NHF). Values are mean±SEM (n=4)

The maximum dose of HAE which did not kill any mice and the minimum dose which led to death of one mouse per group were 6.4 and 3.2 g/kg, respectively. So, LD_50_ of HAE was 4.8 g/kg. As illustrated in Figure 5, none of the HAE concentrations up to 800 μg/ml, could reduce the proliferation of PC12 cells (Figure 5).

**Effect of** ***T. parthenium*** **on motor coordination**

**Figure 6:**
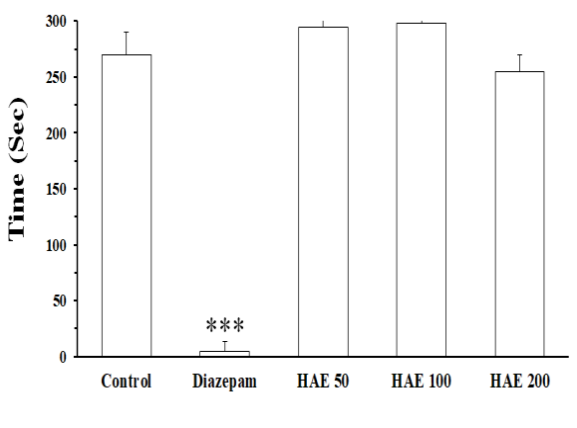
The effects of hydroalcoholic extract of *Tanacetum parthenium* on motor performance in rats. The animals were placed on a rotating rod and the length of time they could maintain their balance on the turntable against the movement’s strength, was recorded. Then, the extract was injected (ip) and after 30 min, the animals were subjected to the test again. Control group received saline containing 1% of Tween 80 as vehicle. Data are mean±SEM of 7 animals in each group. ***p<0.001 vs the control and all three doses of the extract

Rota-rod test results showed no significant differences among the groups when the rats were examined 30 min after injection of the extract. The results showed that diazepam-treated animals maintained their balance on rota-rod apparatus for a significantly shorter period of time (p<0.001) in comparison with the control and extract-treated groups (Figure 6).

## Discussion

In the present study, we evaluated the hypnotic effects of *T. parthenium* for the first time. The results of our study indicated that HAE and the EAF fraction induce hypnotic effects. Also, neurotoxicity test showed that the extract did not affect cell viability. Because HAE did not produce any effect on rota-rod test, it seems that its effects on sleeping time and sleep latency, are not mediated by affecting motor movement. The hypnotic assessment method was based on prolongation of sleep induced by pentobarbital, which is a classic pharmacological method for screening sedative hypnotic agents (Rakhshandah et al., 2010 ▶). In agreement with the previous studies and as expected, diazepam significantly enhanced the sleeping time induced by pentobarbital; these results indicated that our study method was optimized (Emamghoreishi and Heidari-Hamedani, 2015 ▶). Consistently, we observed that pretreatment of mice with flumazenil reversed its effect on sleep. Also, we found that flumazenil inhibits the hypnotic effect of *T. parthenium* extract. 

Many neurotransmitters play a role in regulating sleep behavior. Neurons located in the anterior hypothalamus release GABA to inhibit wake-promoting areas in the hypothalamus and brainstem (Murillo-Rodríguez et al., 2009 ▶; Datta, 2010 ▶). The barbiturate pentobarbital binds GABA receptors.

Benzodiazepines such as diazepam enhance the affinity of GABA for its receptor and thereby increase pentobarbital-induced sleeping time (Awad et al., 2009 ▶). In order to gain a better insight into the nature of the effective compounds responsible for the hypnotic effect of *T. parthenium,* the fractions of HAE were prepared: (1) The WF which contains polar agents and water-soluble constituents of the plant (e.g. glycosides, quaternary alkaloids and tannins); (2) the EAF which contains constituents with intermediate polarity (e.g., some flavonoids); and (3) the NHF that has non-polar agents like sterols, alkanes and some terpenoids. This work showed that EAF was the only fraction which could significantly prolong the sleep duration or reduce the sleep latency. Besides, flumazenil reversed EAF effect on sleep duration (Tian et al., 2011 ▶).

Studies have revealed hypnotic effects of a wide variety of herbal medicine components. These components include alkaloids, terpenoids (e.g. linalool), steroids, and flavonoids (e.g., quercetrin, and luteolin) (Edewor-Kuponiyi, 2013 ▶). This plant has many natural products, including sesquiterpene lactones (eudesmanolides, germacranolides, and guaianolides), but the compounds probably include one or more of the sesquiterpene lactones including parthenolide. Parthenolide is a germacranolide. Other potentially components include flavonoids and volatile oils (Akpulat et al., 2005 ▶; Pareek et al., 2011 ▶).* T. parthenium* contain some flavonoids such as quercetin and luteolin (Williams et al., 1999 ▶; Long et al., 2003 ▶). It was reported that quercetin is able to cross the blood-brain barrier and induce some effects in the central nervous system including neuroprotective and antioxidant actions (Youdim et al., 2004 ▶; Paulke et al., 2006 ▶; Ishisaka et al., 2011 ▶). Kambe et al. (Kambe et al., 2010 ▶) showed that quercetin enhances non-rapid eye movement sleep in dark period in rats.  Luteolin is a widespread flavonoid aglycon that was reported as devoid of specific affinity for benzodiazepine receptor with anxiolytic-like effects (Coleta et al., 2008 ▶). Besides, some studies showed that alpha-pinene derivatives that are found in this plant may possess sedative and mild tranquilizing effects (Pareek et al., 2011 ▶). 

It should be noted that based on the rota-rod assay data, the hypnotic effect is not due to affecting motor movement. The toxicity test exhibited an LD_50_ value of 4.8 g/kg for HAE isolated from *T. parthenium*. This dose is much higher than its hypnotic doses (50-200 mg/kg). Besides, HAE and fractions even at high concentrations, did not reduce viability of neuronal cells. Hence, it seems that hypnotic effects of *T. parthenium* accompanied no neurotoxicity.

In conclusion, the current study showed that *T. parthenium *had significant sedative-hypnotic effects. Isolation and characterization of the active constituent may yield a novel sedative-hypnotic agent.

## Conflict of interest

The authors declare that there is no conflict of interest regarding the publication of this article.

## References

[B1] Akhila JS, Shyamjith D, Alwar M (2007). Acute toxicity studies and determination of median lethal dose. Current science.

[B2] Akpulat HA, Tepe B, Sokmen A, Daferera D, Polissiou M (2005). Composition of the essential oils of Tanacetum argyrophyllum (C Koch) Tvzel var argyrophyllum and Tanacetum parthenium (L) Schultz Bip (Asteraceae) from Turkey. Biochem Syst Ecol.

[B3] Auld F, Maschauer EL, Morrison I, Skene DJ, Riha RL (2017). Evidence for the efficacy of melatonin in the treatment of primary adult sleep disorders. Sleep Med Rev.

[B4] Awad R, Muhammad A, Durst T, Trudeau VL, Arnason JT (2009). Bioassay‐guided fractionation of lemon balm (Melissa officinalis L) using an in vitro measure of GABA transaminase activity. Phytother Res.

[B5] Coleta M, Campos MG, Cotrim MD, de Lima TCM, da Cunha AP (2008). Assessment of luteolin (3′, 4′, 5, 7-tetrahydroxyflavone) neuropharmacological activity. Behav Brain Res.

[B6] Datta S (2010). Cellular and chemical neuroscience of mammalian sleep. Sleep Med.

[B7] Edewor-Kuponiyi TI (2013). Plant-derived compounds with potential sedative and anxiolytic activities. Int Basic Appl Sci.

[B8] Emamghoreishi M, Heidari-Hamedani G (2015). Sedative-hypnotic activity of extracts and essential oil of coriander seeds. Iran J Med Sci.

[B9] Ferreri MC, Gutiérrez ML, Gravielle M C (2015). Tolerance to the sedative and anxiolytic effects of diazepam is associated with different alterations of GABAA receptors in rat cerebral cortex. Neurosci.

[B10] Hosseini A, Sobhanifar MA, Forouzanfar F, Aghaee A, Rakhshandeh H (2018). Hypnotic effect of red cabbage (Brassica oleracea) on pentobarbital-induced sleep in mice. J Pharm Bioallied Sci.

[B11] Ishisaka A, Ichikawa S, Sakakibara H, Piskula MK, Nakamura T, Kato Y, Ito M, Miyamoto K, Tsuji A, Kawai Y (2011). Accumulation of orally administered quercetin in brain tissue and its antioxidative effects in rats. Free Radic Biol Med.

[B12] Jain NK, Kulkarni SK (1999). Antinociceptive and anti-inflammatory effects of Tanacetum parthenium L extract in mice and rats. J Ethnopharmacol.

[B13] Kambe D, Kotani M, Yoshimoto M, Kaku S, Chaki S, Honda K (2010). Effects of quercetin on the sleep–wake cycle in rats: Involvement of gamma-aminobutyric acid receptor type A in regulation of rapid eye movement sleep. Brain Res.

[B14] Khorasani A (1371). Islamic Culture Press Center; Makhzan-al' advieh.

[B15] Long C, Sauleau P, David B, Lavaud C, Cassabois V, Ausseil F, Massiot G (2003). Bioactive flavonoids of Tanacetum parthenium revisited. Phytochem.

[B16] Murillo-Rodríguez E, Arias-Carrión O, Sanguino-Rodríguez K, González-Arias M, Haro R (2009). Mechanisms of sleep-wake cycle modulation. CNS Neurol Disord Drug Targets.

[B17] Pareek A, Suthar M, Rathore GS, Bansal V (2011). Feverfew (Tanacetum parthenium L): A systematic review. Pharmacogn Rev.

[B18] Paulke A, Schubert-Zsilavecz M, Wurglics M (2006). Determination of St Johns wort flavonoid-metabolites in rat brain through high performance liquid chromatography coupled with fluorescence detection. J Chromatogr B.

[B19] Rakhshandah H, Shakeri MT, Ghasemzadeh MR (2010). Comparative hypnotic effect of Rosa damascena fractions and Diazepam in Mice. Iran J Pharm Res.

[B20] Riemann D, Nissen C, Palagini L, Otte A, Perlis ML, Spiegelhalder K (2015). The neurobiology, investigation, and treatment of chronic insomnia. Lancet Neurol.

[B21] Tian S, Shi Y, Zhou X, Ge L, Upur H (2011). Total polyphenolic (flavonoids) content and antioxidant capacity of different Ziziphora clinopodioides Lam extracts. Pharmacogn Mag.

[B22] Vinkers CH, Olivier B (2012). Mechanisms underlying tolerance after long-term benzodiazepine use: a future for subtype-selective GABAA receptor modulators?. Adv Pharmacol Sci.

[B23] Williams CA, Harborne JB, Geiger H, Hoult JRS (1999). The flavonoids of Tanacetum parthenium and T vulgare and their anti-inflammatory properties. Phytochem.

[B24] Youdim KA, Qaiser MZ, Begley DJ, Rice-Evans CA, Abbott NJ (2004). Flavonoid permeability across an in situ model of the blood–brain barrier. Free Radic Biol Med.

